# Global Perspective on Kidney Transplantation in Malaysia

**DOI:** 10.34067/KID.0000000000000510

**Published:** 2024-07-12

**Authors:** Muhammad Iqbal Abdul Hafidz, Hasdy Haron, Mohamad Zaimi Abdul Wahab

**Affiliations:** 1 Department of Nephrology, Universiti Teknologi MARA, Selangor, Malaysia; 2 National Transplant Resource Centre, Kuala Lumpur, Malaysia; 3 Hospital Kuala Lumpur, Kuala Lumpur, Malaysia

**Keywords:** kidney transplantation

## Introduction—High Prevalence of ESKD and Low Rate of Kidney Transplantation

Malaysia is a developing country in Southeast Asia and is classified as an upper-middle-income country. It has the sixth highest prevalence of treated ESKD in the world at 1584 per million population (pmp).^1^ Despite performing its first living donor kidney transplantation in 1975 and its first deceased donor kidney transplantation (DDKT) the following year, Malaysia has one of the lowest rates of kidney transplantation (KT) in the world.^2^ Over the past 10 years, Malaysia performed only 80–180 KT per year (2–5 pmp) with living donor KT making up approximately 70%–80% of the transplants.^3^ This is a brief review of the existing transplant program and organ donation in Malaysia.

## Current Transplant Setup

Access to KT in Malaysia is low because of a lack of transplant centers, transplant surgeons and nephrologists, poor immunology laboratory support, wide availability of hemodialysis, and geographical factors. KT is performed in public hospitals under the Ministry of Health (MOH), university hospitals under the Ministry of Education, and private hospitals. These three health organizations run independently of each other. Only two public hospitals perform KT, both located in the capital city. They are also the only centers performing DDKT in the country. KT is performed by urologists with a special interest in transplant surgery. Currently, there are only two transplant surgeons in these public hospitals performing both the living and deceased transplants. There is no formal training program for transplant surgery in Malaysia. Those who intend to take up transplant train under transplant surgeons until they are deemed ready to perform the surgery. General transplant makes up part of the nephrology subspecialty training. Many of the transplant-trained surgeons and nephrologists who leave the public service to join private practice do not perform KT and prefer the less stressful and more lucrative nontransplant work.

The cost of a KT, including workup and immunosuppression, is minimal in public hospitals, at cost price in university hospitals, and expensive in private hospitals. Unfortunately, there has never been a long-term national strategy particularly integrating the three organizations to develop the field of transplant, and the current situation has remained *status quo*.

## Living Donation

Living donors are defined as first-degree or second-degree relatives and legal spouses. Any donor other than this must be referred to the Unrelated Transplant Approval Committee under the MOH. The National Organ, Tissue, and Cell Transplantation Policy 2007 prohibits the commercialization of organ donation. This policy has not been updated, preventing new approaches like kidney paired donation from taking off. As a result, Malaysia performs ABO-incompatible and HLA-incompatible KT employing different desensitization protocols between centers and achieving relatively good outcomes.

Organ trafficking does not occur in Malaysia. Malaysia is a signatory to the Declaration of Istanbul. However, many patients still travel overseas for commercial transplants in the absence of a donor. This is done through an intermediary introduced by other patients or friends. It is compulsory for the nephrologists treating these patients to register them with the national renal registry. The number of overseas transplants has seen a significant reduction with only 4% of all KT in 2020.^3^ At its peak in 2004, 78% of all KT recorded in Malaysia were performed overseas.

## Deceased Donation and the Low Organ Donation Rate

The existing support for Malaysia's DDKT program, such as infrastructure and transportation, is currently adequate. Legislations and policies, such as the Human Tissues Act 1974 and the Consensus Statement on Brain Death 2003, are also in place to ensure that DDKT is regulated and performed ethically. Despite this, Malaysia remains one of the countries with a very low rate of DDKT. Malaysia performs only about 30–40 DDKTs per year (0.9–1.1 pmp).^3^

The scarcity of kidneys forced the allocation system to be changed. The previous system was solely based on dialysis vintage, leading to issues with cardiovascular disease and vascular calcification in the recipients. In 2012, it was changed to include only patients with an Estimated Post Transplant Survival score<40% and excluded anyone above this. The Estimated Post Transplant Survival, however, is used on its own without matching it to a Kidney Donor Profile Index. This gained criticism as it put patients with diabetes, older age, previous transplants, and long dialysis vintage at a great disadvantage.

The low rate of DDKT stems from the lack of organ donors. This is a result of a complex relationship between beliefs, knowledge, and attitudes that influence not only the public but also the health care providers (HCPs).

## Malaysia's Diversity, Beliefs, Attitudes, and Knowledge

Malaysia has a population of 34.2 million people with very diverse demographics. Ethnically, it is made up of Malays (57.9%), Chinese (22.9%), Indians (6.6%), and many different indigenous groups (12.4%). The Malays are almost all Muslims, Chinese Buddhists, and Indian Hindus while Christianity is also widely practiced. Religion, culture, and superstition shape the belief system of Malaysian populations, and these are often intertwined. Although all the major religions permit organ donation and transplantation, it continues to be the main barrier to organ donation in Malaysia.^4^ This is a consistent finding specifically among Malay Muslims. Despite a national religious ruling (fatwa) issued in 1970 permitting organ donation and transplantation, it has failed to convince the majority of Muslims. Issues related to Islam continue to be raised such as whether it is permissible to donate to a non-Muslim, and *vice versa*, avoiding delay of the burial process, body mutilation, and the importance of being resurrected in the afterlife with an intact body.^5^ As a result, the Malays make up the lowest percentage of donors despite having the highest rate of ESKD and comprising approximately 70% of DDKT recipients.^6^ A common belief unrelated to religion is fear of the organ retrieval process where there is a notion that the deceased can feel pain during the surgery.^7^

Malaysia adopts an opt-in policy where individuals need to register to be organ donors. Attitudes among the public were positive toward being an organ pledger although some declined because of superstitions of inviting bad luck on themselves if they did so.^4,5^ Family approval is an important factor in decision making among Malaysians and is a major reason to decline organ donation.^4,5^ There is a lack of public awareness and understanding of the complex issue of organ donation. Many people did not know how to register to become an organ pledger while the idea of an opt-out system was not well supported.^4,7,8^

## The Role of Health Care Providers

HCPs working in the critical care units are central to the success of the DDKT. Currently, these transplant front liners do not receive formal training in organ procurement and coordination. The National Transplant Resource Center under the MOH was established to coordinate deceased organ transplantation.

Studies showed that the beliefs and attitudes of Malaysia's HCPs toward organ donation were similar to those of the public.^9^ There was an inertia in approaching the family of potential donors, performing brain death examinations, and contacting the National Transplant Resource Center.^9,10^ HCPs were found to have significant gaps in knowledge of organ procurement and coordination and wished for more training in this area.^9^

The MOH changed its approach in 2019 and made 16 major hospitals around Malaysia transplant-focused hospitals. They were required to form an organ procurement unit with full-time clinicians within the hospital. Another 26 hospitals were instructed to employ a full-time transplant coordinator to facilitate the process of organ donation. This resulted in an improvement in deceased donations in some hospitals, but certain major hospitals still failed to provide any donors.

## Current Strategies and Outlook

Current strategies have focused more on the the Malays to dispel religious misconceptions about organ donation and transplantation. Public forums frequently include imams, and awareness messages are given in mosques and through sermons during Friday prayers, which many Muslims attend. Advocacy efforts are aimed at the younger generations, specifically university students, through collaboration with the Ministry of Education and the use of social media.

In 2019, Malaysia joined other Southeast Asia countries in a European Union–funded project led by the University of Barcelona to train HCP in organ donation and coordination. The HCP received training on topics such as brain death, donor management, and family approaches tailored to the local settings.

Another significant step by the government was to introduce a feature on a national health app called MySejahtera for the public to register as an organ donor. This app was initially created specifically to manage the coronavirus disease 2019 pandemic and was compulsory for all adults to hold. The app has now evolved into a one-stop center digital health app.

**Figure 1:**
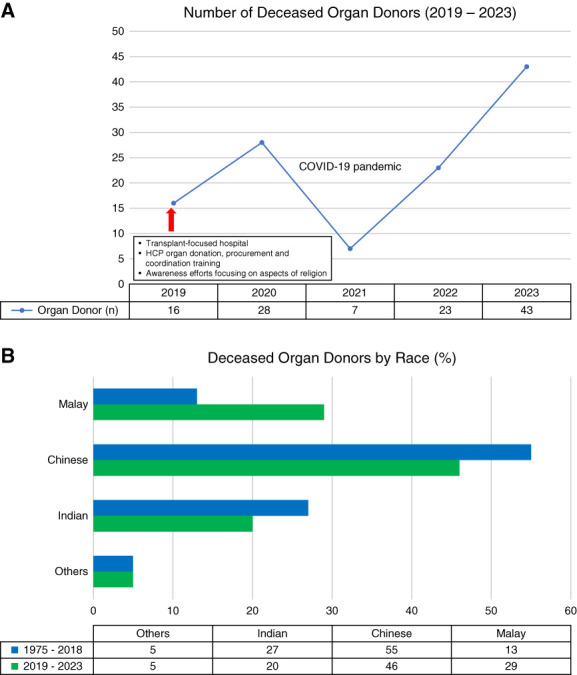
**Improvements in the rate of deceased organ donors nationally and Malay donors after major strategies in 2019.** (A) Number of deceased organ donors. (B) Deceased organ donors by race.

These strategies have seen the number of deceased donors increase over the past 5 years, with the number of Malay donors increasing to 29% (Figure 1).^6^ If this trend continues, Malaysia, as a multiracial, multireligious, and multicultural country, can show similar countries the way to a successful deceased donor program.
